# Effects of high- and moderate-intensity exercise on central hemodynamic and oxygen uptake recovery kinetics in CHF-COPD overlap

**DOI:** 10.1590/1414-431X20199391

**Published:** 2020-02-14

**Authors:** A. Mazzuco, A.S. Souza, W.M. Medeiros, P.A. Sperandio, M.C.N. Alencar, F.F. Arbex, J.A. Neder, A. Borghi-Silva

**Affiliations:** 1 Laboratório de Fisioterapia Cardiopulmonar, Departamento de Fisioterapia, Universidade Federal de São Carlos, São Carlos, SP, Brasil; 2 Setor de Função Pulmonar e Fisiologia Clínica do Exercício, Universidade Federal de São Paulo, São Paulo, SP, Brasil; 3 Laboratory of Clinical Exercise Physiology, Division of Respiratory and Critical Care Medicine, Department of Medicine, Queen's University, Kingston, ON, Canada

**Keywords:** Chronic heart failure, Chronic obstructive pulmonary disease, Oxygen uptake, Heart rate, Recovery kinetics, Exercise test

## Abstract

The oxygen uptake (V˙O_2_) kinetics during onset of and recovery from exercise have been shown to provide valuable parameters regarding functional capacity of both chronic obstructive pulmonary disease (COPD) and chronic heart failure (CHF) patients. To investigate the influence of comorbidity of COPD in patients with CHF with reduced ejection fraction on recovery from submaximal exercise, 9 CHF-COPD male patients and 10 age-, gender-, and left ventricle ejection fraction (LVEF)-matched CHF patients underwent constant-load exercise tests (CLET) at moderate and high loads. The V˙O_2_, heart rate (HR), and cardiac output (CO) recovery kinetics were determined for the monoexponential relationship between these variables and time. Within-group analysis showed that the recovery time constant of HR (P<0.05, d=1.19 for CHF and 0.85 for CHF-COPD) and CO (P<0.05, d=1.68 for CHF and 0.69 for CHF-COPD) and the mean response time (MRT) of CO (P<0.05, d=1.84 for CHF and 0.73 for CHF-COPD) were slower when moderate and high loads were compared. CHF-COPD patients showed smaller amplitude of CO recovery kinetics (P<0.05) for both moderate (d=2.15) and high (d=1.07) CLET. Although the recovery time constant and MRT means were greater in CHF-COPD, CHF and CHF-COPD groups were not differently affected by load (P>0.05 in group *vs* load analysis). The ventilatory efficiency was related to MRT of V˙O_2_ during high CLET (r=0.71). Our results suggested that the combination of CHF and COPD may further impair the recovery kinetics compared to CHF alone.

## Introduction

Exercise intolerance is a multifactorial hallmark of both chronic heart failure (CHF) (1) and chronic obstructive pulmonary disease (COPD) (2). The consequences of the COPD comorbidity in patients with CHF and vice versa are directly linked to imbalances in the oxygen transport pathways, which encompass perfusion and/or diffusion disorders of the cardiovascular, respiratory, muscular, and cerebral systems, characteristics present in the pathophysiological process of both diseases (3).

The oxygen uptake (V˙O_2_) kinetics during onset of and recovery from exercise have been shown to provide valuable parameters regarding functional capacity of both COPD and CHF patients, with submaximal exercise being an important assessment ally as far as its metabolic characteristics come closer to activities of daily living (4,5). Several factors may contribute to the delay in V˙O_2_ kinetics during recovery from exercise, including delayed creatine phosphate restoration (6,7), increased arteriovenous oxygen difference due to lower cardiac output (CO) after exercise (6), and slow restoration of venous blood (7) and replenishment of energy stores in peripheral skeletal muscles (8). All these factors, however, are not correlated to markers of disease severity like left ventricular ejection fraction (LVEF) (6,8). Besides, CHF patients with more severe disease, lower exercise capacity, and a flawed ventilation (V˙_E_) may be identified by impaired CO kinetics in exercise recovery (9). In addition, the rate of recovery of V˙O_2_ at 2 minutes after exercise has been shown to be the strongest prognostic factor of major cardiac events, such as death, heart transplantation, and mechanical heart implantation in severe CHF patients (10).

The reduction in systemic oxygen (O_2_) delivery, the higher O_2_ kinetics during recovery, and the hypoxemia possibly due to hypercapnia in healthy individuals may be explained by the presence of an imposed expiratory flow limitation device during exercise (11). In COPD patients, few studies have explored V˙O_2_, heart rate (HR), and CO from recovery kinetics. A clinical study investigating respiratory gas exchanges and heart rate (HR) kinetics during early-phase recovery after a symptom-limited cardiopulmonary exercise testing (CPET) in COPD patients showed that the quarter-time recovery of V˙O_2_ increased with disease severity and the kinetics in the early recovery period was slowed compared to the control group (12). Recently, the V˙O_2_ recovery kinetics (half-time of V˙O_2_ recovery (T_1/2_ V˙O_2_)) after submaximal exercise has been associated with established parameters of exercise capacity in COPD patients (13). The authors showed that the impairment of the T_1/2_ VO_2_ also depends on disease severity and degree of airflow limitation, suggesting that prolonged dyspnea or leg effort after exercise, commonly experienced by COPD patients, may be understood by differences in recovery ability.

The calculation of recovery kinetics can be obtained by measuring the time constant (s) of the explored variable (V˙O_2_, HR, or CO) by adjusting the curve using a monoexponential equation, where 63% of the time to achieve stabilization of the variable of interest, adjusted by its maximum value in the steady-state exercise (9), represented the τ (s). In addition, the mean response time (MRT) is defined as the sum of the exponential time constant of decay plus a delay term, from end-exercise to the onset of exponential decay (11). Both variables express important diagnostic and prognostic markers to assess the physiological mechanisms underlying exercise intolerance and the impact of COPD on CHF patients.

In this context, previous studies have shown that V˙O_2_ and HR recovery kinetics are slowed in the presence of COPD (12–13) or CHF (8–10) on isolation, as represented by τ and MRT. However, to the best of our knowledge, the recovery kinetics in patients with overlapped CHF and COPD has not been thoroughly explored. Moreover, it is not known whether the kinetics of V˙O_2_, HR, and CO recovery are also intensity-dependent, contrasting the presence of COPD in CHF patients.

Therefore, the main objective of this study was to investigate the influence of comorbidity of COPD in patients with CHF with reduced ejection fraction on V˙O_2_, HR, and CO recovery kinetics after moderate and high constant-load exercise test (CLET). Considering that the combination of these two chronic diseases may potentiate the abnormalities observed in each case individually, we hypothesized that the cardiovascular (represented by the slower response of τ and MRT) and the respiratory interaction (represented by the association with ventilatory efficiency indices) in patients with the coexistence of CHF and COPD may further impair recovery from submaximal intensity exercise (moderate and high), consequently slowing the recovery kinetics to a greater extent compared to patients with CHF alone.

## Material and Methods

### Subjects

Nine sedentary males with a smoking-related spirometric diagnosis of COPD (forced expiratory volume in 1 s (FEV_1_)/forced vital capacity (FVC) <0.7) (14), resting arterial oxygen tension ≥60 mm Hg at room air, and echocardiographic evidence of heart failure with reduced LVEF <40% and 10 age-, gender-, and LVEF-matched CHF patients were enrolled in this prospective, cross-sectional clinical study. All patients were seen by the same cardiologists and pulmonologists at an outpatient specialized clinic and optimally treated before study initiation for at least 3 months (Table 1).

**Table 1:** Baseline demographic and clinical characteristics data of patients with chronic heart failure (CHF) and CHF-chronic obstructive pulmonary disease (COPD).

	CHF (n=10)	CHF-COPD (n=9)
Demographics		
Age, years	61.1±6.7	66.2±5.9
BMI, kg/m^2^	26.2±3.3	24.8±2.9
mMRC ≥2, %	0.7	0.3
NYHA score II-III, %	0.8	0.7
Smoking, never/ex/current, n (%)	0/9 (0.9)/1 (0.1)	0/9 (1.0)/0
pack-years	49.4±26.2	53.9±35.5
Echocardiogram		
LVEF, %	31.0±4.6	34.4±6.6
Lung function		
FVC, % pred	82.8±11.4	79.9±11.3
FVC, L	3.3±0.5	3.0±0.5
FEV_1_, % pred	82.7±12.1	65.7±15.3*
FEV_1_, L	2.5±0.4	1.9±0.5*
FEV_1_/FVC	77.8±3.7	62.5±10.0**
PaO_2_, mmHg	84.5±10.5	75.7±7.6
PaCO_2_, mmHg	32.8±3.5	35.3±1.9
SpO_2_, %	95.5±2.3	94.9±1.8
Main co-morbidities, n (%)		
Hypertension	9 (90)	8 (88.9)
Type II diabetes	6 (60)	2 (22.2)
Hypercholesterolemia	6 (60)	6 (66.7)
Sleep apnea	3 (30)	1 (11.1)
Chronic kidney disease	0	3 (33.3)
Chronic atrial fibrillation	1 (10)	1 (11.1)
CAD	4 (40)	2 (22.2)
Alcoholism, ex/current	3 (30)/0	2 (22.2)/0
Therapies, n (%)		
LABA	0	4 (44.4)*
LAMA	0	2 (22.2)
ICS	0	1 (11.1)
Xanthines	0	1 (11.1)
LABA + LAMA	0	3 (33.3)
LABA + ICS	0	2 (22.2)
LABA + ICS + LAMA	0	0
Beta-blockers	10 (100)	9 (100)
ACE inhibitors/ARB II	10 (100)	8 (88.9)
Calcium channel blocker	0	3 (33.3)
Diuretics	10 (100)	9 (100)
Digitalis	4 (40)	4 (44.4)
Platelet antiaggregants	5 (50)	6 (66.7)
Statins	7 (70)	6 (66.7)
Antiarrhythmics	1 (10)	1 (11.1)
Hypoglycemics	6 (60)	2 (22.2)
Gastric protection drugs	5 (50)	3 (33.3)

BMI: body mass index; mMRC: modified Medical Research Council; NYHA: New York Heart Association; LVEF: left ventricular ejection fraction; FVC: forced vital capacity; FEV_1_: forced expiratory volume in 1 s; PaO_2_: partial pressure for O_2_; PaCO_2_: partial pressure for CO_2_; SpO_2_: peripheral oxygen saturation; CAD: coronary arterial disease; LABA: long-acting beta_2_-agonist; LAMA: long-acting anticholinergics; ICS: inhaled corticosteroids; ACE inhibitors: angiotensin-converting-enzyme inhibitors; ARB II: angiotensin II receptor blockers. LVSD-n=9 for CHF and 6 for CHF-COPD; LAV-n=9 for CHF and 6 for CHF-COPD; n patients were able to achieve acceptable test criteria for DL_CO_=9 for CHF and 7 for CHF-COPD. Data are reported as means±SD or n (percentage). *P<0.05; **P<0.001 (Student’s *t*-test).

No decompensation episodes occurred in any enrolled subject for at least one month prior to study initiation. Main exclusion criteria were long-term O_2_ therapy (for at least 6 months), recent (within 6 months) rehabilitation, type I or non-controlled type II diabetes mellitus, and peripheral vascular disease, orthopedic/rheumatological/neurological conditions that would preclude participation in the study, other concomitant respiratory diseases, any contraindication to exercise testing according to the American Heart Association guidelines (15), and inability to understand and cooperate with the procedures. All subjects were informed about study objectives, experimental procedures, and potential risks and gave written informed consent before study initiation. The study was approved by the Medical Ethics Committee of São Paulo Hospital, Brazil (protocol 424.135/2013) and Ethics Committee on Human Research at the Federal University of São Carlos (UFSCar), Brazil (protocol 515.654 /2014).

### Experimental design

All patients underwent a comprehensive evaluation divided into three different days with intervals of one week between them. On day 1, patients underwent a clinical evaluation by a physician and a physical therapist, followed by lung function tests and Doppler echocardiography. On the second day, patients underwent a CPET. After 48 h, patients were invited to conclude 2 CLETs with intervals of 30 min between them. Moderate intensity CLET (40–50% of maximal) was applied first and after a rest period of 4 min, high intensity CLET (70–80% of maximal) was applied. The order used is based on previous studies that consider that moderate intensity (40–50% of peak workload) does not affect subsequent intensity (16,17).

### Day 1

#### Lung function

Spirometry (1085 ELITE DTM, Medical Graphics Corporation, USA) was measured according to American Thoracic Society/European Respiratory Society guidelines (18). Resting blood gases were obtained by samples from the radial artery (19).

#### Doppler echocardiography

Echocardiography was performed by the same trained echocardiographist (20) and the left ventricular ejection fraction (%) was determined.

#### Clinical and physical therapy evaluation

Patients were evaluated by a clinician to assess disease stability and check the use of all mediations and general condition. An experienced physical therapist evaluated the patients' functional capacity by a report of physical limitation and applied a physical exam in order to evaluate musculoskeletal disorders that could interfere with the tests to be applied.

### Day 2: maximal exercise testing to determine submaximal loads

#### Cardiopulmonary exercise testing

Patients performed a symptom-limited, ramp incremental exercise test (5 or 10 W) on an electronically braked cycle ergometer (Corival 400, Lode BV, Netherlands). V˙O_2_ (mL/min), carbon dioxide output (V˙CO_2_; mL/min), minute ventilation (V˙_E_; L/min), end-tidal partial pressures for O_2_ and CO_2,_ respiratory exchange ratio (RER=V˙CO_2_/V˙O_2_), tidal volume (VT, mL), and respiratory frequency (breaths/min) were measured breath by breath using a computer-based system (CardiO2 System, Medical Graphics). All ventilatory and cardiocirculatory variables of gas exchange were averaged every 15 s, and V˙O_2_ peak was defined as the highest value achieved during the test (21). HR (beats/min) was determined using the R-R intervals from a 12-lead electrocardiogram and continuously monitored potential cardiac arrhythmias. Peripheral oxygen saturation was determined by pulse oximetry (SpO_2_ in %; POX 010-340, Mediaid, USA]. Patients were also asked to rate their “shortness of breath” at exercise cessation using the 0-10 Borg's category ratio scale (22). In addition, using CPET data, the V˙_E_/V˙CO_2_ relationship from the beginning of exercise to peak exercise was derived by least squares linear regression (i.e., y=x + b, m=slope) (Microsoft Excel, Microsoft Corp., USA). The oxygen uptake efficiency slope (OUES) was calculated using the log-transformation (base 10) of V˙_E_ (L/min) on the x-axis and V˙O_2_ (L/min) on the y-axis (23). Circulatory power (mmHg^.^mL O_2_/kg/min) was defined as the product of peak V˙O_2_ and peak SBP (24). Exercise ventilatory power (mmHg) was defined as peak SBP divided by the V˙_E_/V˙CO_2_ slope (25). Heart rate (bpm) was determined using the RR interval from the Polar¯ system (Polar¯ S810i, Finland).

### Day 3: experimental protocol

One week after CPET, patients performed a two-stage cycle ergometer test starting at 40 to 50% during 4 min of exercise and a 4-min passive resting period. After this period, a second intensity was applied at 70–80% of the peak work rate achieved on CPET (high) during 4 min or until the limit of tolerance. All ventilatory, cardiocirculatory, and metabolic variables were also obtained.

#### Cardiac output

Hemodynamic variables (stroke volume: SV (mL), HR (bpm), and CO (L/min)) were non-invasively assessed throughout the CLET by a calibrated signal-morphology impedance cardiography system (PhysioflowPF-5^TM^, Manatec Biomedical, France). Principles of operation and algorithms have been previously described (26).

#### Kinetics analysis

The breath-by-breath V˙O_2_, HR, and hemodynamic CO data were time-aligned to 30 s of the end of exercise bouts and interpolated second by second. The kinetics of these responses were determined by non-linear regression using a least squares technique and the exponential time constant τ of V˙O_2_, HR, and CO decay in recovery was determined for the monoexponential relationship (4) between these variables and time during 240 s of recovery using the following formula (SigmaPlot 11.0, Systat Software Inc., USA): Y = Y0 – A * (1 – e^−(t−Td)/τ^), where Y is the V˙O_2_, HR, or CO, Y0 is V˙O_2_, HR, or CO at time zero (beginning of the recovery phase), A is V˙O_2_, HR, or CO amplitude during exercise recovery (mL·kg^-1^·min^-1^, bpm, L/min, respectively), Td is the time delay (s), and τ is the exponential time constant (s). This time constant reflects the time required to achieve 63% of the difference between starting and baseline values. The overall kinetics was determined by the mean response time (MRT= τ+Td). Fit quality was determined by the sum of the squared residuals and by the coefficient of determination R^2^.

### Statistical analysis

Results are reported as means±SD unless otherwise stated. All statistical analyses were conducted at a 95% level of significance using SPSS Statistics for Windows software package, Version 17.0 (IBM, USA). After visual inspections, the Shapiro-Wilk test was used to verify the normality of data and the Levene's test was used to check homogeneity of data. For baseline patient characteristics and responses of CPET, CHF and CHF-COPD patients were contrasted by independent *t*-tests or Mann-Whitney test, according to variable distribution. Fisher's exact test was used for categorical data comparisons. Two-way analysis of variance (ANOVA) for repeated measures was performed contrasting intensities as within-subjects variable (moderate and high) and groups (CHF and CHF-COPD) as between-subjects factor to compare peak cardiopulmonary, metabolic, hemodynamic, and recovery kinetics responses with Bonferroni's *post hoc* test. For respective *post hoc* observations, we determined Cohen's d effect size for the differences between groups and intensities and interpreted as small (0.20–0.49), medium (0.50–0.79), large (0.80–1.29), and very large (>1.30) (27).

Pearson's correlation coefficients were used to test the association between τ and MRT of VO_2_, HR, and CO (dependent variables) and markers of disease severity at rest (LVEF and VEF_1_) and during maximal exercise (peak V˙O_2_, peak HR, HR recovery at 1 min post-CPET-HRR_1_, Δ V˙O_2_/Δ work rate (WR), OUES, and V˙_E_/V˙CO_2_ slope) (independent variables). The magnitude of correlations was determined considering the following classification scheme for r values: 0.26–0.49: low or weak; 0.50–0.69: moderate; 0.70–0.89: strong or high; 0.90–1.0: very high (28).

## Results

### General characteristics

**Figure 1:**
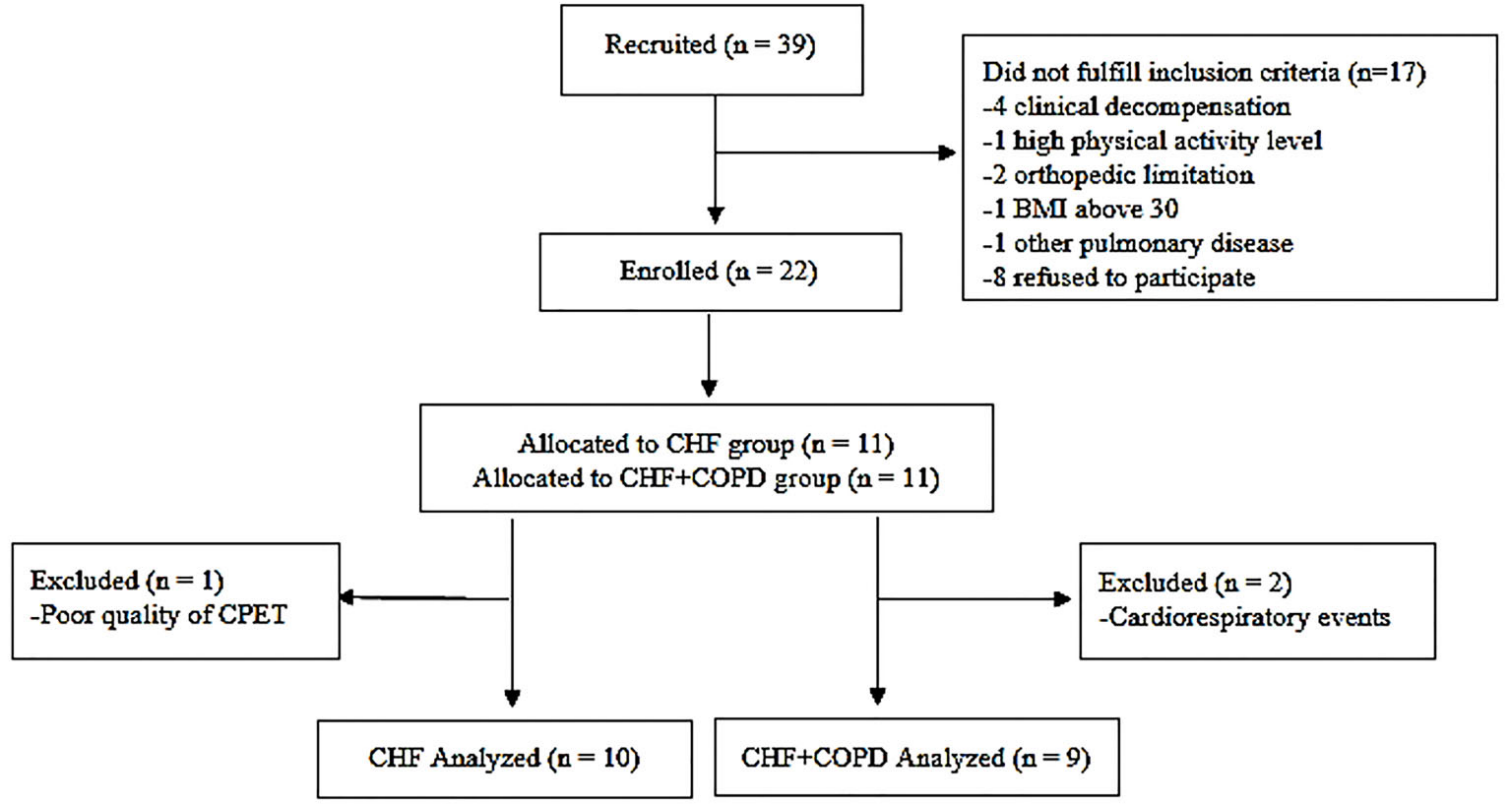
Flowchart of the study. COPD: chronic obstructive pulmonary disease; CHF: chronic heart failure.

Figure 1 shows the flowchart of the study. A total of 39 patients (19 with CHF and 20 with CHF-COPD) were recruited from a specialized outpatient CHF/CHF-COPD clinic (convenience sample). Seventeen did not fulfill inclusion criteria [clinical decompensation (1 CHF, 3 CHF-COPD), high physical activity level (1 CHF), orthopedic limitation (1 CHF, 1 CHF-COPD), BMI above 30 (1 CHF-COPD), pulmonary fibrosis (1 CHF-COPD), and refusal to participate (5 CHF, 3 CHF-COPD)]. Eleven patients with CHF and 11 with CHF-COPD were initially included, however 3 were excluded [did not perform CPET adequately (1 CHF) and cardiorespiratory events (2 CHF-COPD, 1 respiratory arrest and 1 sustained ventricular tachycardia both during CLET)].

Baseline demographic and clinical characteristics are shown in Table 1. Subjects were matched for age, BMI, symptoms, and LVEF. As expected, CHF-COPD patients presented with lower FEV_1_ and higher long-acting beta_2_-agonist use, however, with non-significant differences in arterial oxygenation and PaCO_2_. The most common cause of CHF was ischemic cardiomyopathy (9 CHF, 5 CHF-COPD), followed by idiopathic etiology (1 CHF, 3 CHF-COPD) and valvular etiology (1 CHF-COPD).

Table 2 shows the comparisons of the cardiopulmonary exercise testing between the groups. The groups presented similar reduced workload on peak and when compared to the predicted values. In addition, groups were similar in relation to all cardiorespiratory and metabolic parameters, and limiting symptom at peak exercise. In both groups, predicted HR were reduced and the groups used beta blockers in similar percentages (see Table 1).

**Table 2:** Comparison of cardiorespiratory and metabolic responses obtained at peak of cardiopulmonary exercise testing between chronic heart failure (CHF) and CHF-chronic obstructive pulmonary disease (COPD) patients.

	CHF (n=10)	CHF-COPD (n=9)
Peak WR, W	71.7±29.7	58.9±14.2
Peak WR, % pred	57.1±19.5	53.4±11.1
Peak V˙O_2_, mL·kg^-1^·min^-1^	15.5±5.0	14.7±2.4
Peak V˙O_2_, % pred	63.5±18.2	63.6±7.7
Rest P_ET_CO_2_, mmHg	31.2±2.6	30.6±3.3
Peak P_ET_CO_2_, mmHg	30.7 [23.8-34.7]	31.6 [28.7-32.4]
Peak RER	1.2 [1.1-1.3]	1.1 [1.0-1.2]
Peak HR, bpm	118.6±20.1	101.7±21.3
Peak HR, % pred	74.5±12.3	66.0±13.2
Peak SBP, mmHg	118.9±23.9	124.7±17.9
Peak DBP, mmHg	70.8±11.3	71.7±13.0
Peak O_2_ pulse, mL·beat^-1^	10.0±3.4	10.4±2.3
Peak SpO_2_, %	97.0±1.6	94.9±3.6
Peak-rest SpO_2_, %	1.0 [(-1.0)-1.0]	0.0 [(-1.0)-0.5]
Peak Borg dyspnea score	7.2±2.5	7.8±1.6
Peak Borg fatigue score	8.5 [6.3-10.0]	10.0 [7.5-10.0]
HRR^1^, bpm	18.8±11.9	10.7±7.2
Δ V˙O_2_/Δ WR, mL·min^-1^·W^-1^	9.3±2.9	8.6±1.9
V˙_E_/V˙CO2 slope	39.5±10.4	37.0±6.2
OUES	1.4±0.5	1.4±0.3
CP, mmHg·mlO2·kg^-1^·min^-1^	1876.7±790.8	1830.1±383.0
EVP, mmHg	3.3±1.2	3.4±0.5

WR: work rate; V˙O_2_: oxygen uptake; P_ET_CO_2_: end-tidal partial pressure for CO_2_; RER: respiratory exchange ratio; HR: heart rate; SBP: systolic blood pressure; DBP: diastolic blood pressure; SpO_2_: pulse oximetry; HRR_1_: heart rate recovery at 1 min; V˙_E_: ventilation; OUES: slope efficiency uptake oxygen; CP: circulatory power; EVP: exercise ventilatory power. Data are reported as mean±SD or median [interquartile range]. No difference was observed between groups (Student’s *t*-test).

### HR, VO_2_, and CO off-kinetics responses to moderate and high CLET

The cardiovascular, ventilatory, and hemodynamic responses to moderate and high intensities CLET are shown in Table 3. In the present study, as expected, in both groups, high intensities led to higher V˙O_2_, V˙CO_2_, HR, VE, and CO (P<0.05). However, peak HR and CO were lower in CHF-COPD group compared with CHF alone when high intensities were applied (P<0.05). There was no interaction between the groups when the two intensities were contrasted (P>0.05).

**Table 3:** Peak responses to moderate and high intensities during constant workload exercise testing in chronic heart failure (CHF) and CHF-chronic obstructive pulmonary disease (COPD) groups.

	Moderate		High		Group	Intensity	Interaction
	CHF	CHF-COPD	CHF	CHF-COPD	P	P	P
V˙O_2_, mL·kg^-1^·min^-1^	10.2±2.6	9.8±1.3	13.4±3.8	13.0±3.0	0.69	0.004	0.99
V˙CO_2_, mL·min^-1^	685.7±191.3	606.3±105.2	976.8±256.9	845.1±190.5	0.06	<0.001	0.92
P_ET_CO_2_, mmHg	27.5±3.3	27.6±3.0	24.9±3.6	25.3±3.6	0.76	0.12	0.98
P_ET_O_2_, mmHg	115.7±5.8	113.6±8.0	120.7±6.0	117.6±10.0	0.18	0.16	0.97
HR, bpm	92.7±12.4	89.4±14.6	115.3±27.3	97.3±18.1	0.01	0.03	0.39
SBP, mmHg	115.2±21.0	115.4±10.3	125.2±25.4	130.2±17.8	0.68	0.06	0.71
DBP, mmHg	74.6±16.7	66.7±12.0	74.6±16.4	70.7±15.3	0.24	0.69	0.68
V˙_E_, L	30.0±6.2	27.7±5.4	46.8±9.1	41.3±11.2	0.11	<0.001	0.85
SV, mL	72.9±11.8	77.2±20.9	80.4±15.3	75.3±21.8	0.69	0.86	0.66
CO, L·min^-1^	6.6±1.5	6.2±2.2	9.1±1.9	7.5±2.5	0.02	0.01	0.57
HRR_1_, bpm	15.0±6.0	13.3±9.4	18.9±11.3	15.4±10.7	0.41	0.33	0.77

V˙O_2_: oxygen uptake; V˙CO_2_: carbon dioxide output; P_ET_CO_2_: end-tidal partial pressure for CO_2_; P_ET_O_2_: end-tidal partial pressure for O_2_; HR: heart rate; SBP: systolic blood pressure; DBP: diastolic blood pressure; V˙_E:_ ventilation; SV: stroke volume; CO: cardiac output; HRR_1_: heart rate recovery in the first minute. Data are reported as mean±SD (ANOVA).

The HR, V˙O_2_, and CO off-kinetics variables at moderate and high exercise intensities in both groups are reported in Supplementary Table S1. There was a significant main effect of intensity on the baseline (Y0) and A of HR, V˙O_2_, and CO in both groups (P<0.05). However, A of CO was lower in CHF-COPD compared with CHF, both at moderate and high intensities (P<0.05).

**Figure 2:**
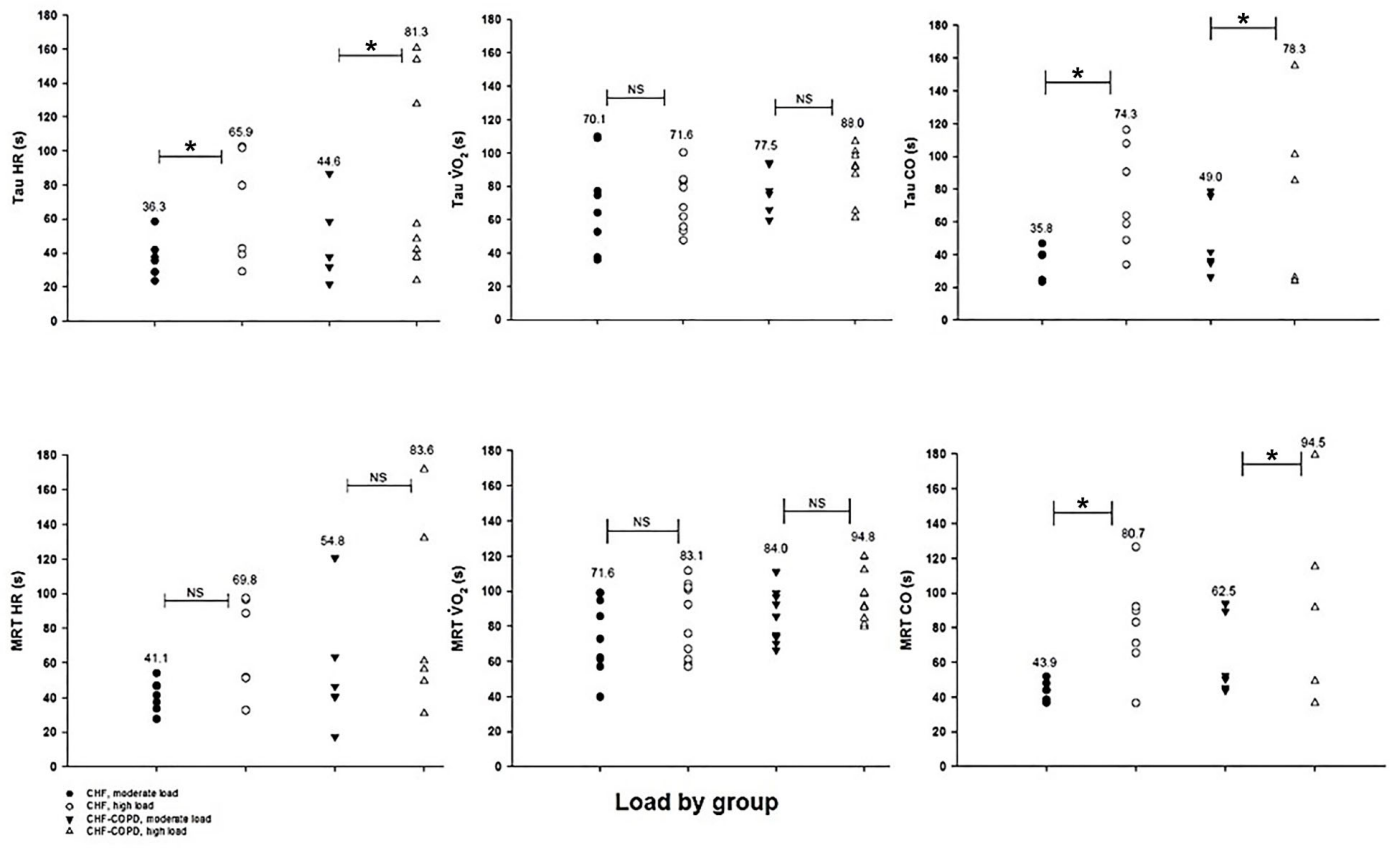
Heart rate (HR), oxygen uptake (V˙O_2_), and cardiac output (CO) off-kinetics variables at moderate and high constant-load exercise test in chronic heart failure (CHF) and CHF-chronic obstructive pulmonary disease (COPD) groups. MRT: mean response time; Tau: time constant; NS: not significant. *P<0.05 (ANOVA) (n=6-8 per group).

Regarding the effects of moderate and high CLET on the exponential time constant τ, there was a significant main effect of intensity on HR and CO recovery kinetics (P<0.05). However, there was a non-significant main effect of group on the τ of these variables. Only the MRT of CO was significantly affected by intensity in both groups (P<0.05). Nevertheless, there was a non-significant interaction between groups versus intensities (Table 3 and Figure 2).

**Figure 3:**
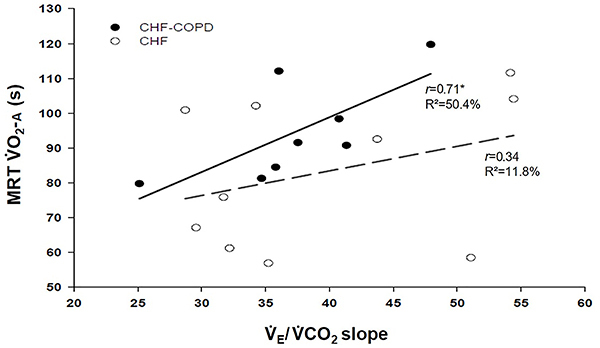
Relationship between ventilation/carbon dioxide output (V˙_E_/V˙CO_2_) slope and mean response time (MRT) of oxygen uptake (V˙O_2_) during high constant-load exercise test in patients with chronic heart failure (CHF) and CHF-chronic obstructive pulmonary disease (COPD). *P<0.05.

As shown in Figure 3, the V˙_E_/V˙CO_2_ slope was significantly related to MRT of V˙O_2_ during recovery from high CLET in CHF-COPD patients but not in CHF.

## Discussion

This is the first cross-sectional study to investigate the influence of comorbidity of COPD in patients with CHF with reduced LVEF on recovery kinetics after moderate and high CLET. Our preliminary analysis showed that the amplitude of HR, VO_2_, and CO were higher at high intensities, regardless of the presence or absence of COPD in CHF. In addition, τ of HR and CO, and MRT of CO were slowed down at high intensities, regardless of the presence or absence of COPD in CHF. However, the amplitude of CO was significantly lower in patients with COPD coexistence in CHF compared with CHF alone, at both moderate and high exercise intensity (P<0.05). Altogether, the recovery kinetics analysis after submaximal exercise (moderate and high loads) might be useful to assess metabolic characteristics that can clinically reflect prolonged dyspnea and leg effort experienced during activities of daily living of CHF and particularly of CHF-COPD patients. Additionally, the assessment of recovery profile of CHF with comorbidity of COPD might also be helpful to determine the effects of exercise interventions such as interval exercise training.

We expected that recovery kinetics after high intensity exercise was statistically greater compared to moderate when within-group contrasts were considered (represented by τ of HR and CO, and MRT of CO). In COPD patients, the off-MRT response of CO was independent of exercise modality, which was attributed to the fact that cycling (at 75% of workload) at moderate intensities and 6-min walking test are submaximal intensities and are maintained at a constant rate of physical effort (29). The two exercise bouts performed in the present study (40-50% and 70-80% of workload), although characteristically submaximal, differed in terms of baseline (Y0) and A of HR, VO_2_, and CO in both groups. Indeed, we observed that differences were more evident in CHF patients, mainly because SV, HR, and consequently CO responses to high-load exercise were attenuated in CHF-COPD patients (Table 3). In fact, it has been shown that effort-independent variables like OUES and τ of VO_2_ kinetics during recovery from submaximal constant-load exercise are clinically useful for detecting training effects in CHF patients (12-week combined cycle interval and muscle resistance training program) (30). In CHF patients who underwent a 12-week high-intensity interval training (4×4 min at 85-95% of peak V˙O_2_), the V˙O_2_ recovery kinetics after submaximal exercise was accelerated by 20% due to improvement in microvascular O_2_ delivery-to-utilization matching once the skeletal muscle deoxygenation during submaximal exercise was attenuated and no changes were observed in CO kinetics (31).

Regarding CO recovery kinetics, results from the current study showed a statistically significant main effect of group on A of CO during recovery both in moderate and high intensity exercise bouts. The between-group effect size calculation revealed very large (d=2.15) and large (d=1.07) effect sizes for moderate and high loads, respectively. Considering that patients in CHF-COPD group reached lower values of CO at peak of exercise at high intensity (CHF=9.1±1.9 *vs* CHF-COPD=7.5±2.5 L·min^-1^, Table 3, P<0.05), it was expected that they would also exhibit lower values of A (CHF=5.4±2.3 *vs* CHF-COPD=3.4±1.4 L·min^-1^, Supplementary Table S1, P<0.05). Myers et al. (9) analyzed the CO recovery kinetics by bioreactance after symptom-limited maximal exercise testing on treadmill and found that CHF patients with more severe disease, lower exercise capacity, and inefficient ventilation also demonstrated slower CO kinetics in recovery compared to controls.

Moreover, in our sample, CHF-COPD patients with abnormal MRT of V˙O_2_ during recovery after high-load exercise also demonstrated inefficient ventilation as evidenced by the strong positive relationship between V˙_E_/V˙CO_2_ slope and MRT of V˙O_2_ during high CLET (P<0.05, r=0.71, R^2^=50%) (Figure 2). A heightened V˙_E_/V˙CO_2_ slope is a hallmark of more severe CHF (32) and in the presence of COPD comorbidity, the expected alterations in lung function further worsen the ventilation-perfusion mismatch in the lungs and the ventilatory efficiency in CHF patients (3). In fact, the results of a recent study indicated that lower maximal exercise capacity in CHF-COPD patients was associated with greater ventilatory inefficiency, attenuated dynamic hyperinflation and lower peak P_ET_CO_2_ compared to patients with COPD alone (33). Thus, our data suggested that the poorer ventilatory efficiency in CHF-COPD patients also reflected impaired V˙O_2_ recovery kinetics particularly after high-CLET performance.

The lack of statistical significance in τ and MRT of HR, V˙O_2_, and CO during recovery showing that CHF and CHF-COPD groups were not differently affected by exercise intensity may be attributed to the small sample size. However, CHF-COPD τ and MRT means were greater than τ and MRT means in the CHF group, showing slowed recovery kinetics in CHF patients with comorbidity of COPD at both moderate and high loads (Supplementary Table S1). Besides, the between-group τ and MRT effect size calculation of HR, V˙O_2_, and CO recovery kinetics showed that differences were more pronounced for the moderate load (effect size >1.3; Supplementary Table S1). In CHF, the delayed recovery from exercise may clinically contribute to the sensation of leg muscle fatigue reported by patients in activities of daily living (34). Following submaximal exercise, the O_2_ recovery kinetics seems to be limited more by O_2_ delivery than O_2_ utilization (35), suggesting that recovery from this type of exercise is limited by a reduced muscle blood flow in CHF patients (36). Recently, a prospective study assessed the role of central and peripheral impairments in the O_2_ transport pathway in limiting exercise tolerance in patients with COPD and CHF overlap compared to FEV_1_-matched COPD and LVEF-matched CHF patients. Besides the fact that patients with CHF-COPD had lower endurance exercise tolerance they also presented a greater impairment in leg muscle blood flow, but not arterial oxygenation, than CHF alone (37). Although in the present study peripheral hemodynamics was not assessed, we speculated that during recovery from exercise bouts, the impairment of muscle blood flow can also contribute to slow the HR, V˙O_2_, and CO recovery kinetics particularly in CHF-COPD patients at higher loads. In addition to mechanisms that lead to reduced O_2_ delivery in CHF patients (heart failure per se, elevated vasoconstriction due to excessive sympathetic activity, higher plasma angiotensin levels, impaired nitric oxide-mediated vasodilation, and blunted redistribution of blood flow from the non-exercise tissues to exercising muscles (4)), the pulmonary mechanics abnormalities in COPD patients may entail negative cardiocirculatory effects (38). The excessive increase in abdominal, pleural, and alveolar pressures can reduce venous return, lowering right ventricle preload and elevating the afterload of both ventricles. Moreover, hyperinflation leads to capillary and pulmonary arteriole compression and may cause mechanical compression in the cardiac chambers decreasing their complacency (38).

### Clinical implications

This is a novel cross-sectional study that aimed to investigate the influence of comorbidity of COPD in patients with CHF with reduced LVEF on recovery kinetics after moderate and high CLET. Our results added relevant information regarding recovery from constant load exercise assessment since the metabolic characteristics of recovery from submaximal exercise can clinically reflect prolonged dyspnea and leg effort experienced in activities of daily living of CHF (4) and very likely of CHF-COPD patients. Despite the lack of statistical significances, the effect size calculation – which is independent from sample sizes (27) – showed large/very large effect sizes in a considerable number of variables. Additionally, the assessment of recovery profile of CHF with comorbidity of COPD might also be useful to determine the effects of intervention that target the increase of skeletal muscle O_2_ delivery and/or reduction of O_2_ demand, such as physical training (31), non-invasive ventilation (39), and/or medicine prescription (e.g., sildenafil) (40).

### Limitations

Certainly, the main limitation of our study was its small sample size. However, CHF and CHF-COPD patients were carefully examined by the joint force of cardiologists and pulmonologists and optimally treated before study initiation. Thus, we are confident that our results provide a good scenario to the alarming consequences of comorbidity of COPD in CHF patients. In addition, we acknowledge that the current results can only be applied in men, which might limit the external validity of the present study. Nevertheless, the homogeneity of our sample increased the internal validity of our results. We also recognize that the addition of a control group might have added comparative analysis to our results.

### Conclusion

This is the first study to investigate the recovery kinetics after submaximal exercise in CHF patients with comorbidity of COPD. Although CHF and CHF-COPD groups were not affected differently by exercise intensity, the amplitude of CO was lower in CHF-COPD at both intensities, suggesting that the combination of CHF and COPD may further impair skeletal muscle O_2_ delivery compared to CHF alone. In addition, the poorer ventilatory efficiency in CHF-COPD patients also reflected impaired V˙O_2_ recovery kinetics particularly after high-intensity exercise performance. Given that patients with chronic cardiac and pulmonary diseases like CHF and COPD experience prolonged dyspnea and leg effort in activities of daily living, the recovery profile assessment after submaximal constant load exercise testing might be helpful to comprehend the metabolic characteristics that contribute to symptoms and exercise intolerance and to determine the effects of interventions such as exercise training, especially in patients with CHF and COPD overlap.

## Supplementary Material

Click here to view [pdf].
